# Super Enhancers in Cancers, Complex Disease, and Developmental Disorders

**DOI:** 10.3390/genes6041183

**Published:** 2015-11-09

**Authors:** Adrienne R. Niederriter, Arushi Varshney, Stephen C. J. Parker, Donna M. Martin

**Affiliations:** 1 Medical Scientist Training Program, Medical School, University of Michigan, Ann Arbor, MI 48109, USA; E-Mail: aniede@med.umich.edu; 2 Department of Human Genetics, Medical School, University of Michigan, Ann Arbor, MI 48109, USA; E-Mails: arushiv@umich.edu (A.V.); scjp@umich.edu (S.C.J.P.); 3 Department of Computational Medicine and Bioinformatics, Medical School, University of Michigan, Ann Arbor, MI 48109, USA; 4 Department of Pediatrics and Communicable Diseases, Medical School, University of Michigan, Ann Arbor, MI 48109, USA

**Keywords:** super-enhancer, stretch enhancer, transcription, cancer, complex disease, developmental disorder

## Abstract

Recently, unique areas of transcriptional regulation termed super-enhancers have been identified and implicated in human disease. Defined by their magnitude of size, transcription factor density, and binding of transcriptional machinery, super-enhancers have been associated with genes driving cell differentiation. While their functions are not completely understood, it is clear that these regions driving high-level transcription are susceptible to perturbation, and trait-associated single nucleotide polymorphisms (SNPs) occur within super-enhancers of disease-relevant cell types. Here we review evidence for super-enhancer involvement in cancers, complex diseases, and developmental disorders and discuss interactions between super-enhancers and cofactors/chromatin regulators.

## 1. Defining Super-Enhancers

Examination of genome-wide association studies (GWAS) has led to the proposal that a large portion of disease-associated genomic variation lies in *cis*-regulatory regions. Classic enhancers were initially defined in SV40 by their ability to modify gene expression in an orientation and position-independent manner by recruiting coactivators and RNA Polymerase II to target genes [1]. More recently, criteria have been established to define super-enhancers as unique areas of the genome that are large, densely bound by transcription factors/cofactors and are thought to play a critical role in defining cell identity by regulating nearby cell-type specific genes [2]. Using chromatin immunoprecipitation followed by sequence analysis (ChIP-seq) to examine binding patterns of master regulators of pluripotency (Oct4, Sox2, Nanog) in mouse embryonic stem cells (ESCs), Whyte *et al.* [2] first characterized super-enhancers as clusters of enhancers that were enriched in both size and binding of the Mediator complex by an order of magnitude or more compared to so-called typical enhancers. These super-enhancers were also noted to have increased occupancy of the ESC transcription factors Klf4 and Esrrb, further supporting their role in maintaining ESC identity.

**Figure 1:**
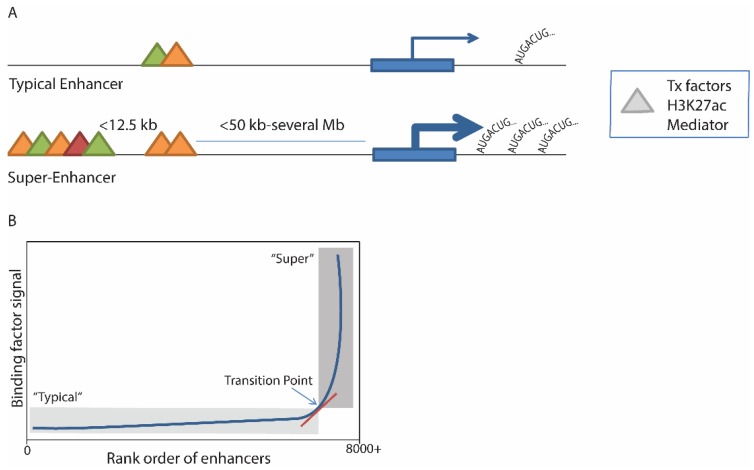
(**A**) Schematic representation of a typical enhancer and a super-enhancer. Definition of super-enhancers is based on identification of various bound transcription (Tx) factors, H3K27ac marks, or Mediator (triangles) and assembly of similar regions within 12.5 kb; (**B**) Distinction of enhancers from super-enhancers is accomplished by ranking enhancers in order of factor binding density, and mathematically identifying the point where the signal begins to rapidly increase; enhancers above this point are considered “super”. For more details, see Table 1. Tx = transcription.

The original description of a super-enhancer used mouse ESCs and was based on a step-wise strategy: (1) identify enhancers as genomic regions bound by master transcription factors (Oct4, Sox2, Nanog in ESCs); (2) stitch together “constituent” enhancer regions within 12.5 kb of each other with similar binding patterns; and then (3) identify a subset of those genomic regions with increased Med1 binding [2]. The separation of super-enhancers from typical enhancers was accomplished by plotting all enhancers in rank order of increasing Med1 signal. A clear point could then be seen whereby transcription factor occupancy begins to increase rapidly, above which enhancers are considered to be “super” (Figure 1). About 40% of Med1 binding signal localized to super-enhancers, which made up less than 3% of total enhancer regions across the genome, due to region size and density of binding [2].

Definitions of super-enhancers vary between ESCs and differentiated cells. In differentiated cells, lineage-specific master regulators are used in place of the master transcription factors Oct4, Sox2, and Nanog to generate binding plots [2]. For example, PU.1, the master transcription factor of pro-B cells, correlates with Mediator binding, suggesting that super-enhancers identified in this manner overlap with those identified using Mediator. This observation allowed for use of another method published around the same time that relies on Med1 only [3]. These frameworks have since been applied to a wide variety of cell and tissue types. Once a super-enhancer is identified, the gene it controls can also be inferred. Relying on proximity, Whyte *et al.* identified the closest transcription start site to a typical super-enhancer, considering that most enhancer looping interactions occur within a distance of 50 kb [2,4]. In ESCs, many genes near super-enhancers have been found to control ESC identity, including Oct4, Sox2, and Nanog, suggesting autoregulation of master transcription factor expression. Additionally, super-enhancer-related genes are expressed at higher levels in ESCs than those near typical enhancer regions [2]. Knockdown of Oct4 or Mediator by shRNA in ESCs also exerts more pronounced effects on super-enhancer related genes, resulting in loss of ESC-specific gene expression and impaired differentiation, suggesting greater sensitivity to perturbation than typical enhancers. These properties of super-enhancer-related gene expression (autoregulation, high-level expression, and sensitivity to perturbation) also hold true for more differentiated cell lineages (myotubes, pro-B cells, T-helper cells, macrophages) and their respective master transcription factors and identity-related genes [2].

## 2. Controversy over Enhancer Characterization

Since their initial characterization by Whyte and Loven in 2013 [2,3], dozens of publications have cited super-enhancers, although the means by which they are identified has been inconsistent (see Table 1). There has also been confusion about specific properties used to define super-enhancers and other secondary characteristics, such as additional chromatin marks and cofactors bound to these regions.

The definition, novelty, and potential misuse of the term super-enhancers were recently discussed in a perspective essay by Pott and Lieb [5]. They argued that super-enhancers are arbitrarily defined (*i.e.*, there is no functional significance to the cutoff between super- and typical-enhancers) and display previously known properties of enhancers. Notably, prior to the classification of super-enhancers, clusters of open regulatory elements (COREs) were described as associated with tissue-specific transcription factors, further lessening the novelty of super-enhancer terminology [6,7]. Use of the term “enhancer” has also shifted from a functional definition of a DNA element that activates transcription of genes from a distance, to a looser definition based on chromatin profiles characterized by DNAse I hypersensitivity, p300 binding, or H3K4me or H3K27ac marks. The recent explosion in large-scale genomic data has led many researchers to carefully revisit these concepts.

**Table 1:** Comparison of markers used to define super-enhancer features.

ChiP-seq Enhancer Identification Strategy	Factor to Distinguish Typical and Super Enhancers	Cell/Tissue Type	Reference
(1) Oct4, Sox2, Nanog (2) Stitch together (3) Med1	Med1	mESC	[2]
		mESC	[8]
(1) Med1 (2) Stitch together		MM1.S cell line SCLC cells Glioblastoma cells	[3]
		Multiple AML cell lines	[9]
(1) H3K27ac (2) Stitch together (3) Med1		Brain Lung Heart Adrenal Monocytes Th cells B cells Hematopoietic stem cells Spleen Small intestine Sigmoid colon Adipose	[10]
(1) PU.1 (2) Stitch together	PU.1	mPro-B cells	[2]
(1) MyoD (2) Stitch together	MyoD	mMyotubes	[2]
(1) T-bet (2) Stitch together	T-bet	mT-helper cells	[2]
(1) C/EBPα (2) Stitch together	C/EBPα	mMacrophages	[2]
(1) EBNA2 (2) Stitch together	EBNA2	EBV-transformed lymphoblastic cells	[11]
(1) H3K27ac (2) Stitch together	H3K27ac	CRC line HCT-116 ER+ line MCF-7	[8]
		Jurkat cells	[9]
		MOLM-1 cells	[12]
		Medulloblastoma cells	[13]
		MYCN-amplified Kelly cells SH-SY5Y cells	[14]
		SCLC cells GLC16 cells NCI-H69 cells NCI-H82 cells	[14]
		mT-cells	[15]
	H3K27ac	EBV-transformed lymphoblastic cell lines	[11]
	Not described	mStriatum	[16]
(1) BRD4 (2) Stitch together	BRD4	Ly1 DLBCL cell line	[17]
		NF-κB activated endothelial cells	[18]

Colors denote studies using similar criteria to differentiate super-enhancers from typical enhancers. Blue = Mediator binding, green = cell-type specific transcription factor, red = H3K27ac, purple = other chromatin cofactor.

Other marks such as H3K27ac, H3K4me1, DNAse hypersensitivity, and p300 have also been explored to delineate super-enhancers [2,10]. In particular, use of H3K27ac (a histone mark delineating active from poised enhancers) to identify super-enhancers in mouse ESCs yielded over three times as many as those originally identified by Med1 signal, over three-fourths of which were unique [2,19]. While H3K27ac binding does not adequately identify all super-enhancers, it is often used as a surrogate for Med1 binding. In another study, twice as many regions were identified as super-enhancers, with more than two-thirds being unique (as compared to Med1 binding) [10]. Interestingly, use of p300 binding as a super-enhancer criterion leads to a higher portion of super-enhancers that overlap with those bound by Med1 [10]. However, p300 ChIP-seq data are not yet available for a wide variety of cell types, limiting its current use as a genome-wide super-enhancer identifier [10].

In an attempt to standardize these approaches, Hnisz and colleagues composed a program which performs a similar stepwise approach to that described by Whyte and Loven (*i.e.*, stitching together constituent enhancers or similar individual enhancer elements within 12.5 kb (at least 2 kb from a TSS), ranking the stitched enhancers by H3K27ac (or other) signal, then mathematically identifying the transition point to separate super-enhancers from typical enhancers) [2,3,10]. This program, termed ROSE (Rank Ordering of Super-Enhancers), is rapidly becoming the most employed method, due in part to free access to the program and wide availability of H3K27ac data (Table 1). Multiple methods of identifying super-enhancers have been used, as Med1 binding data are often unavailable and cell-type-specific transcription factors are not always known. Nonetheless, the lack of consistent methodologies makes comparison between studies difficult.

Another recent term, “stretch enhancer”, was used by Parker *et al.* to denote long, non-stitched (≥3 kb) genomic regions with specific chromatin marks [20]. Specifically, an integrative analysis of epigenomic profiles in the form of ChIP-seq data for five histone marks (H3K27ac, H3K4me1, HeK4me3, H3K27me3 and H3K36me3) in nine human cell types was performed. Recurrent patterns of combinations of these marks were identified and used to segment the genome into “chromatin states” using a multivariate hidden Markov model as implemented in the ChromHMM software package [21,22]. Enhancer segmentations ≥3 kb in length were classified as stretch enhancer states; whereas the median length of typical enhancers was observed to be 800 bp. These stretch enhancer regions have been shown to be tissue and cell type specific and contain transcription factor binding motifs enriched for tissue relevant disease associated variants or quantitative traits [20,23].

**Figure 2:**
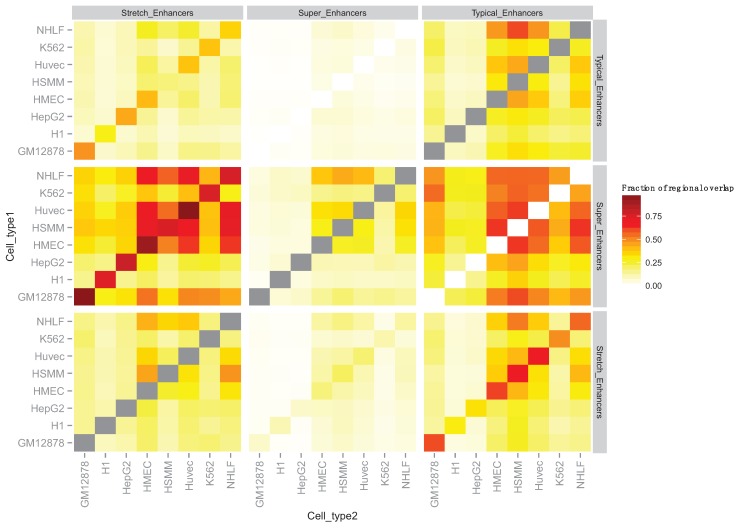
Regional overlap across enhancer classifications in eight matched cell types. Fraction of enhancers of the y-axis facet (cell type 1) that contain overlaps with enhancers of the x-axis facet (cell type 2) for each of the cell types. Note that fractional overlaps are calculated using total counts from cell type 1 in the denominator. Grey tiles denote overlaps of an element with itself.

To compare stretch enhancers with super enhancers and typical enhancers (based on the super enhancer definition), we first complied the number of typical and super enhancers [10] and stretch enhancers [20] reported for nine matched cell types (Table 2). Next, we computed the fraction of regional overlap between all three enhancer classifications across the eight cell types (Figure 2). This allowed us to precisely measure the extent of overlap between all the different enhancer classes, which shows that super enhancers are generally a subset of stretch enhancers in a matched cell type. For example, in human GM12878 cells, 257 super-enhancers and 10,355 stretch enhancers were identified, and 249 overlap between the two classes (see Figure 2: lower left cell in the heatmap from column 1, row 2, fractional overlap = 0.97). Conversely, due to a difference in counts between super and stretch enhancers (Table 2), super enhancers make up only a small fraction of stretch enhancers in a matched cell type (for example in GM12878, see Figure 2: lower left cell in the heatmap from column 2, row 3, fractional overlap = 0.02). There is observable overlap between stretch enhancers and typical enhancers, whereas super and typical enhancers were reported as a disjoint set (Figure 2). The level of overlap across non-matched cell types (Figure 2, off the diagonal) is generally less than the level of overlap across different enhancer classes within a matched cell type.

Super-enhancers, as well as stretch enhancers, also overlap with DNA methylation valleys (large stretches of DNA with reduced methylation, often near developmentally-important genes) and locus control regions (regulatory elements controlling specific genes) [5,20]. This overlap between super-enhancers and other identified large-scale regulatory regions suggests they may be functionally or conceptually equivalent, with differences arising from the methods used to classify them [5].

**Table 2:** Number of stretch [20], super and typical enhancers [10] reported in eight matched cell types.

	Enhancer Type/Cell Type	GM12878	H1	HepG2	HMEC	HSMM	Huvec	K562	NHLF
1	Stretch Enhancers	10355	6426	7969	12997	7284	10890	10142	9858
2	Super Enhancers	257	684	497	1099	1029	912	742	784
3	Typical Enhancers	10358	6322	5512	17024	23869	16572	11281	13263

## 3. Super-Enhancer Functions

Some studies have attempted to determine super-enhancer functionality. Hnisz *et al. e*valuated super-enhancer functions individually and combinatorially by cloning individual constituents of mouse ESC super-enhancers into luciferase reporter vectors [8]. Interestingly, functional interaction of three constituents of the *Pou5f1* (*OCT4*) super-enhancer, or individual elements stitched together, were neither additive nor synergistic; rather, they had a complicated interdependence on each other’s activity, with optimal transcriptional activity resulting from the presence of all constituents. Use of clustered regularly spaced interspaced short palindromic repeats (CRISPR/Cas9) to delete individual constituents led to reduced expression of the nearby associated genes, consistent with lack of redundant functions between constituents. Important functional data on super-enhancers also come from previous chromatin interaction analyses showing that constituents physically interact more frequently than in typical enhancers [24]. Clearly, additional studies with this level of rigorous analysis are necessary to carefully explore super-enhancer functions.

Multiple enhancers can contribute to gene regulation in a variety of ways, through differential activation during particular developmental stages at specific loci, or via redundant, additive, or synergistic regulation of gene expression. While some super-enhancers may exhibit these known functions, additional complex interactions contribute to their proposed roles in defining cell identity. Further distinction of super-enhancers from typical enhancers emphasizes the ability of their constituents to interact and function as a unit.

Some known functional connections exist between super-enhancers and signaling pathways. In particular, the terminal transcription factors of Wnt (TCF3), TGF-β (SMAD3), and LIF (STAT3) signaling pathways show increased binding to super-enhancers, and manipulation of these developmental pathways leads to profound changes in super-enhancer related genes [8,10]. Key cell identity genes may have evolved the clustered topology present in super-enhancers in order to allow signaling pathways to regulate genes that control cell identity during development and also contribute to disease. For example, TCF4, the terminal transcription factor of the Wnt pathway, is enriched in super-enhancers of oncogenes in a human colorectal cancer cell line, and modulation of Wnt tends to result in corresponding changes in expression of genes associated with super-enhancers. Moreover, super-enhancers can be gained or lost as a unit in various cancers, strengthening the notion that they act in unison. Taken together, these data suggest that super-enhancers display characteristics that are functionally unique from those of typical enhancers. It remains to be seen whether a more precise functional definition of super-enhancers can be made which would clarify the qualities that distinguish them from typical enhancers, stretch-enhancers, and regulatory regions currently defined primarily by physical characteristics.

**Figure 3:**
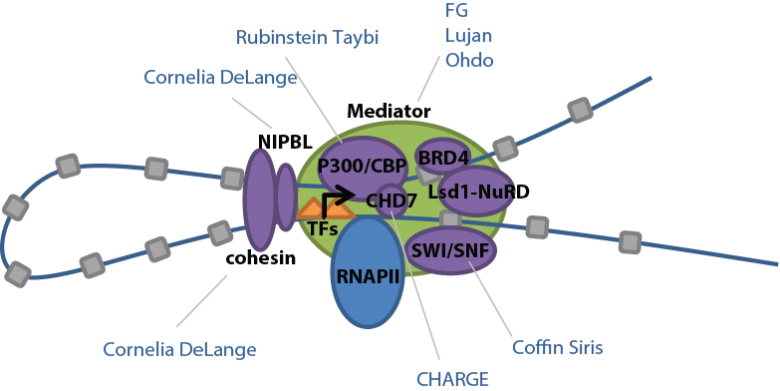
Schematic of transcriptional factors found in ESC super-enhancers and their related developmental diseases. Adapted from [10] with permission.

Interestingly, in addition to high levels of Mediator, super-enhancers have also been shown to harbor increased levels of other cofactors and chromatin regulators, such as histone acetyltransferases [10]. These additional bound factors are known to interact and ultimately regulate gene expression, likely contributing to the high level of transcriptional activity noted at super-enhancers. The Mediator complex acts as a central hub for transcriptional regulation, interacting with other complexes and factors to play roles in epigenetic modification, DNA loop formation, transcriptional initiation, elongation and termination, and RNA processing [25]. For example, Mediator binds Nipbl, which assists in loading and unloading of cohesin to facilitate looping of enhancers to promoters (Figure 3) [26].

Mediator also interacts with p300 and CREB binding protein/CBP (closely related coactivators and acetyltransferases which facilitate relaxation of chromatin), BRD4 (which binds to Mediator and acetylated histones to regulate RNAPII elongation), Brg1 (a component of the SWI/SNF chromatin remodeling complex), and the Lsd1-NuRD complex (which functions in the decommissioning of enhancers) (Figure 3) [10]. Overlapping ChIP-seq binding profiles for these components suggest that they form a large complex at super-enhancers, thereby influencing transcription in a coordinated fashion. This co-regulation also likely explains why mutations in these cofactors lead to overlapping clinical features in humans (see Mendelian Disease section).

Complex interactions between components of this large complex and constituent enhancers all contribute to their transcriptional regulation of cell identity and function via developmental signaling pathways. Many super-enhancer associated genes undergo loss of expression upon loss of ESC differentiation [14,16,17,27], leading to speculation that super-enhancers may facilitate cell state transitions during development [10]. The enrichment of super-enhancers at genes that are developmentally regulated also means super-enhancers are inherently vulnerable to perturbation and disease.

## 4. Super-Enhancers in Mendelian Disease

A wide variety of congenital disorders result from disruption of proper transcriptional processes during embryogenesis (for general review of transcriptional dysregulation in human disease see [28]). Interestingly, germline mutations in nearly all cofactors that exhibit enriched binding at super-enhancers have been associated with developmental disorders, many of which have overlapping phenotypes (Table 3). Mutations in *MED12*, the major component of Mediator Complex, cause several identifiable syndromes in humans, including FG, Lujan, and Ohdo Syndromes (Table 3). These disorders are phenotypically distinct yet share the common underlying mechanism(s) of disrupted *MED12* dosage or function.

**Table 3:** Developmental disorders linked to cofactors bound to super-enhancers as identified by [10].

Syndrome	Facial Abnormalities	Skeletal Abnormalities	Organ Abnormalities	ID	Behavior	Other	Gene(s)
CHARGE	Square-shaped facies	Many	Eye Heart Ear	Y	Autistic features		*CHD7*
Coffin Siris	Coarse facies	Hypoplasia of fingertips/toes		Y			*ARID1A* *ARID1B* *SMARCA4* *SMARCB1* *SOX11*
Cornelia de Lange	Arched/joined brows Long eyelashes Low set ears Small, spaced teeth Small, upturned nose	Short stature	Eye Heart Ear	Y	Autistic features		*NIPBL* *SMC1A* *SMC3*
FG	Prominent forehead	Broad thumbs, toes		Y	ADHD		*MED12*
Lujan	Macrocephaly High nasal root Short philtrum Narrow palate Crowded teeth Micrognathia	Long fingers, toes		Y	ADHD Aggressive Shy	Hypotonia	*MED12*
Ohdo	Narrow palpebral fissures Ptosis Broad nasal bridge Long philtrim Rounded nose Narrow palate	Fifth finger clinodactyly	Ear	Y		Hypotonia Seizures	*MED12*
Rubenstein-Taybi	Down-sloping palpebral fissures Hypoplastic maxilla	Short stature Broad thumbs, toes	Eye Heart Kidney	Y		Speech difficulties	*CREBBP* *EP300*

ID = intellectual disability; Y = Yes.

Defective cohesin or NIBPL results in autosomal dominant Cornelia de Lange syndrome (CdLS), a complex developmental disorder characterized by a constellation of abnormalities including growth and developmental delays, upper limb malformations, microcephaly, craniofacial dysmorphisms, and other structural birth defects. Loss of one copy of various components of the Mediator-cohesin complex can also lead to transcriptional dysregulation and disease, suggesting complex dosage sensitive effects.

In addition to cohesin and NIPBL, mutations in *AFF4*, a component of the super-elongation complex, were recently identified in children with phenotypes overlapping with CdLS [29]. ChiP-seq binding profiles are similar between fibroblasts of children with this new syndrome (called CHOPS) and CdLS [29]. AFF4, RNAPII and cohesin physically interact, suggesting that CdLS and CHOPS exhibit disrupted transcriptional elongation as a common molecular pathogenesis [29]. RNAPII and cohesin also bind heavily at super-enhancers, suggesting the super-elongation complex is also present in high levels at super-enhancers [10]. By extension, dysregulation of super-enhancer mediated transitions between cell fates may contribute generally to developmental disorders, although this has yet to be proven. Additionally, disrupted function of super-enhancers may help explain the polygenic nature of many related developmental disorders.

Mutations in many different transcription factors and chromatin remodeling proteins lead to developmental disorders in humans. CHARGE Syndrome, a multiple congenital anomaly condition caused by heterozygous mutations in the chromatin remodeler *CHD7*, exhibits widely variable clinical expressivity and penetrance of craniofacial anomalies, vision and hearing losses, cardiac malformations, and neuronal developmental defects in both peripheral and central nervous systems [30]. CHD7 binds to thousands of sites in the mammalian genome, and exhibits co-occupancy with other proteins such as EP300 in embryonic stem cells [31,32]. Interestingly, heterozygous mutations in *EP300* and the closely related *CREBBP* cause Rubenstein-Taybi syndrome, characterized by microcephaly, broad thumbs and toes, and characteristic craniofacial anomalies [33]. The transcription factor *SOX11*, mutated in Coffin-Siris syndrome, was also recently identified as a downstream genetic target of *CHD7* [34]. Coffin-Siris Syndrome exhibits craniofacial dysmorphisms and characteristic digit anomalies; however, mutations in several other genes, including the Swi2/Snf2-related DNA-dependent ATPases *ARID1A*, *ARID1B*, *SMARCA4* and *SMARCB1* also cause Coffin-Siris, suggesting broad genetic heterogeneity [35]. It is tempting to speculate that commonalities between these various developmental disorders relate to similar underlying molecular mechanisms such as super-enhancer dysfunction. However, it is equally likely that overlapping temporal and spatial properties of gene expression, modulation of downstream signaling pathways, and tissue-specific factors play important roles.

## 5. Super-Enhancers in Complex Disease

Much work thus far has focused on undifferentiated ESCs, yet the vast array of differentiated cell types and their associated diseases may also be targets of super-enhancers. To date, super-enhancers have been implicated in complex hematological, endocrine, and autoimmune disorders. Not surprisingly, evidence for association between super-enhancers and common complex diseases came from analysis of the distribution of 5303 trait-associated SNPs identified by GWAS [10]. Most (93%) SNPs are located in non-coding regions, 64% of which occur in enhancer regions (~1/3 of the genome as denoted by H3K27ac) with disproportionate enrichments in super-enhancers compared to typical enhancers. As expected, SNPs associated with specific diseases tend to occur in disease-relevant cell types, especially those SNPs located in super-enhancers.

Enrichment of SNPs in super-enhancers occurs in Alzheimer Disease, Type 1 Diabetes, and Systemic Lupus Erythematosus. Super-enhancers contain 5 of 27 (19%) disease associated SNPs in brain tissue, 13 of 67 (19%) disease associated SNPs in T-helper-cells, and 22 of 67 (33%) disease associated SNPs in B-cells [10]. Similar enrichments have also been noted in rheumatoid arthritis, multiple sclerosis, systemic scleroderma, primary biliary cirrhosis, Crohn’s disease, Graves’ disease, vitiligo, inflammatory bowel disease, type 1 diabetes, and atrial fibrillation [10,15]. Recent studies of the epigenomic landscape of pancreatic islet cells also found enrichment of GWAS SNPs associated with Type 2 Diabetes in “stretch” enhancers [20,23]. Clearly, further research is required to better understand the functional impact of super-enhancer related SNPs in specific disease states.

Although the precise mechanisms that control the inflammatory response are poorly understood, many complex diseases exhibit features of underlying chronic inflammation. To further dissect how pro-inflammatory signaling contributes to pathogenesis, Brown *et al.* explored the epigenomic dynamics of activated endothelial cells [18]. Upon stimulation of endothelial cells with the archetypal pro-inflammatory stimulus TNFα, the transcriptional regulator NF-κB localized genome-wide to enhancers and promotors where it is known to engage in cross-talk with chromatin remodeling machinery such as BRD4. Interestingly, exceptionally high levels of BRD4 were found in a small number of enhancers, essentially establishing a new set of super-enhancers. These super-enhancers are located proximal to genes (such as cytokine *CCL2*) that contribute to the inflammatory response, whereas genes associated with resting endothelial cell states were found to have reduced BRD4 binding [18]. These observations demonstrate that stimulus-driven master transcription factors such as NF-κB can induce rapid transcriptional responses through massive redistribution of super-enhancer binding factors that then promote transcription.

While previous studies have largely investigated associations between SNPs and enhancers or transitions between active super-enhancers, loss of super-enhancers in disease-associated tissues has also been observed. Achour *et al.* showed that in mouse models for Huntington Disease (HD), RNAPII and H3K27ac are preferentially decreased in the brain striatum, a region known to exhibit extensive transcriptional misregulation in humans with HD [16]. Super-enhancers enriched in mouse striatal tissues regulate genes that control neuronal identity and function, such as Gata2 [16]. The proposed mechanism for decreased H3K27ac marks in the HD striatum is progressive alteration of the acetyltransferase CBP leading to super-enhancer destabilization [16]. If true, this mechanism could provide insights into mechanisms for related neurodegenerative disorders that are also linked to transcriptional dysregulation.

## 6. Cancer

Tumorigenesis was first associated with super-enhancers shortly after their characterization in 2013, when Loven *et al.* began investigating why cancer therapies targeting ubiquitous chromatin regulators result in specific genetic effects on tumor cells [3]. In particular, selective inhibition of *c-MYC* transcription was attained by inhibition of BRD4, a ubiquitous bromodomain chromatin remodeling protein that recruits the positive transcription elongation factor P-TEFb, thereby regulating transcriptional elongation. Because the multiple myeloma cell line used to study the effects of BRD4 inhibition contains a rearrangement resulting in *MYC* expression driven by an *IgH* enhancer, Loven *et al.* hypothesized that features of the *IgH* enhancer are responsible for the target selectivity of BRD4 inhibition. BRD4 displays similar binding patterns to Mediator, localizing to regulatory regions of actively transcribed genes, especially super-enhancers. Unsurprisingly, these regions are associated with genes having a role in multiple myeloma biology, including *c-MYC*. In addition, BRD4 inhibition results in preferential loss of BRD4 at super-enhancers, resulting in a corresponding decrease in MED1 binding and transcription. Together, these observations support the hypothesis that MYC inhibition is attained by BRD4 depletion at its enhancers [36].

Interestingly, comparison of multiple myeloma tumor cells and related healthy cells suggests that cancer cells “acquire” specific super-enhancers near oncogenes, since they tend to occur in a gene desert near *c-MYC* yet are absent in healthy cells [10]. This acquisition of specific super-enhancers presumably results from chromosomal translocation, gene amplification, increased transcription factor expression, or somatic mutation of noncoding elements, all of which are known to contribute to tumorigenesis. Interestingly, genomic rearrangements can also contribute to tumorigenesis by simultaneous ectopic transcriptional activation and haploinsufficiency during super-enhancers transitions [12]. These new insights into the mechanisms of selective inhibition of oncogenic drivers provide critical information for cancers with increased *MYC* expression including multiple myeloma, Burkitt’s lymphoma, acute myeloid leukemia, and acute lymphoblastic leukemia. In particular, for multiple myeloma it is estimated that nearly half of cases carry a *MYC* rearrangement and that most of these reposition *MYC* near a super-enhancer [37].

**Figure 4:**
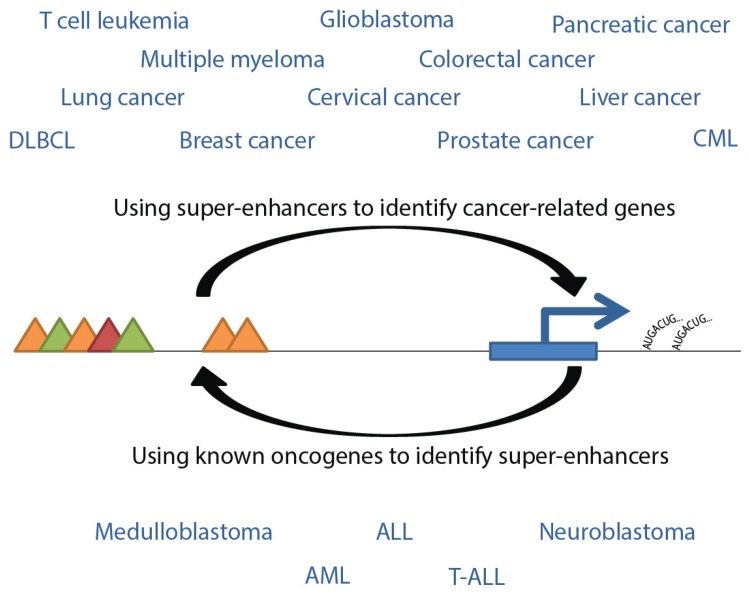
Interpretive flow of super-enhancers and genes related to cancers. Listed are cancer types for which gene-associated super-enhancers have been identified, either using super-enhancer sequences or oncogenes as the reference point. Triangles represent transcription factors, post-translational modifications, or Mediator complex, as in Figure 1.

Many studies have been conducted to investigate cancer associations with super-enhancers and can be generally divided largely into two categories: those using super-enhancers to identify related genes which may play a role in cancer biology, and those identifying super-enhancers that drive known oncogenes (Figure 4). Super-enhancer related cancers include pediatric tumors such as glioblastoma [3,10], medulloblastoma [27], neuroblastoma [14], and T-ALL [10,27,38]. Together, these tumors are expected to account for more than half of new pediatric cancer cases in 2015 [39]. See Table 4 for selected genes associated with pediatric cancer super-enhancers. In addition, numerous high-incidence adult cancers have been associated with super-enhancer dysfunction, including cancers of the breast [8,10], colon [8,10], lung [3,10,14], prostate [10] and pancreas [10], and various leukemias [9,10,12]. Characterization of super-enhancer related cancers may provide useful mechanistic information underlying the development and progression of tumors, and may reveal mechanisms useful for treatments that overcome cellular heterogeneity. In particular, targeting of oncogenic signaling pathways and related super-enhancers may be effective ways to regulate growth of tumor cells dependent on transcription.

**Table 4:** Selected Pediatric Cancer Super-Enhancers and their implicated genes.

Cancer Type	Implicated Genes	Reference	
Glioblastoma	*BHLHE40* *FOSL2* *RUNX1*	[3]	
	*CCND1* *CDK6* *EGFR* *JUN* *MET* *MYC* *NOTCH2*	[10]	
Medulloblastoma	*GFI1* *GFI1B*	[13]	
Neuroblastoma	*MYCN*	[14]	
T-ALL	*BCL11A* *BCL6* *CCND3* *CDK6* *IKZF1* *MYB* *MYC* *NOTCH1* *NOTCH2* *RUNX1* *SHH* *TAL1* *TERT* *TRIB1*	[10]	
	*TAL1*	[38]	
	*MYC*	[27]	

Whole-genome sequencing has also resulted in the identification of variants in putative enhancer regions, which may be super-enhancers. Recently, the mutational load of patients with CLL was expanded to include mutations in enhancer regions enriched for both lymphocyte-specific transcription factor binding sites and histone marks related to enhancer elements only in a lymphoblastoid B-cell line. This region was found to exert control over *PAX5*, an essential player in B-cell differentiation. CRISPR/Cas9-based genome editing of this region demonstrated that inactivation of the enhancer could be achieved through point mutation or deletion, resulting in a 40% reduction in the expression of *PAX5* [40].

## 7. Progress toward Treatment

Associations between super-enhancers and cancers, together with the observation that super-enhancers are sensitive to the amount of bound transcription factors and coactivators, led to the use of inhibitors to reduce super-enhancer transcriptional effects. JQ1, a BET bromodomain inhibitor, reduced BRD4 occupancy at super-enhancers (97% reduction *vs.* 71% reduction in typical enhancers [3]), reduced levels of MED1 binding, increased RNA polymerase pausing, and depleted mRNA in super-enhancer related genes such as *MYC* [3,11], *OCA-B* [17], *BCL6* [17], and *EVI1* [12]. In *in vitro* models of inflammation, JQ1 treatment also suppressed rolling and adhesion properties of leukocytes, and transmigration of neutrophils. Further, JQ1 treatment in *in vivo* models of murine atherosclerosis attenuated early atherogenesis [18]. These studies are promising, but it is also worth noting that JQ1 treatment results in growth suppression and genome wide loss of BRD4 (up to 70%) raising concerns about its target specificity [3]. A similar BET inhibitor, I-BET, has also been investigated as a treatment for super-enhancer related cancer with similar results [9].

In addition to BET inhibitors, other treatments have targeted cyclin-dependent kinases that regulate RNAPII initiation and elongation. One such inhibitor targets CDK7, which primarily affects transcripts with short half-lives such as anti-apoptosis and cell cycle regulators [41]. Use of the covalent CDK7 inhibitor THZ1 in neuroblastoma and small cell lung cancer cells results in significant reduction of active transcripts, especially those associated with super-enhancers, most notably the *MYC* family [14]. Together, these studies demonstrate that targeting the cell’s gene expression program at super-enhancers can result in selective gene inhibition, especially for genes responsible for cancer processes, and can lead to strong and selective cytotoxicity.

While these methods of potential therapy have targeted transcription machinery acting at super-enhancers, another therapeutic approach has been proposed to target genes associated with super-enhancers. In particular, the enrichment of STAT at super-enhancers suggested that use of Tofacitinib, an upstream inhibitor of JAK, would have a targeted influence on super-enhancers [15]. Indeed, Tofacitinib had a larger effect on transcript levels of genes controlled by super-enhancers compared to typical enhancers [15]. In particular, it is not surprising that Tofacitinib, used to treat rheumatoid arthritis, disproportionately affects transcription of rheumatoid arthritis risk genes [15]. This provides new knowledge to the mechanism of action for a drug that has been on the market for several years. Furthermore, this is another exciting example of specific targeting of genes contributing to disease using knowledge gained by super-enhancer studies, and suggests that new therapeutics may be developed in order to act at other disease-related super-enhancers.

## References

[1] Banerji J., Rusconi S., Schaffner W. (1981). Expression of a β-globin gene is enhanced by remote SV40 DNA sequences. Cell.

[2] Whyte W.A., Orlando D.A., Hnisz D., Abraham B.J., Lin C.Y., Kagey M.H., Rahl P.B., Lee T.I., Young R.A. (2013). Master transcription factors and mediator establish super-enhancers at key cell identity genes. Cell.

[3] Loven J., Hoke H.A., Lin C.Y., Lau A., Orlando D.A., Vakoc C.R., Bradner J.E., Lee T.I., Young R.A. (2013). Selective inhibition of tumor oncogenes by disruption of super-enhancers. Cell.

[4] Sanyal A., Lajoie B.R., Jain G., Dekker J. (2012). The long-range interaction landscape of gene promoters. Nature.

[5] Pott S., Lieb J.D. (2015). What are super-enhancers?. Nat. Genet..

[6] Song L., Zhang Z., Grasfeder L.L., Boyle A.P., Giresi P.G., Lee B.K., Sheffield N.C., Graf S., Huss M., Keefe D. (2011). Open chromatin defined by DNasei and FAIRE identifies regulatory elements that shape cell-type identity. Genome Res..

[7] Gaulton K.J., Nammo T., Pasquali L., Simon J.M., Giresi P.G., Fogarty M.P., Panhuis T.M., Mieczkowski P., Secchi A., Bosco D. (2010). A map of open chromatin in human pancreatic islets. Nat. Genet..

[8] Hnisz D., Schuijers J., Lin C.Y., Weintraub A.S., Abraham B.J., Lee T.I., Bradner J.E., Young R.A. (2015). Convergence of developmental and oncogenic signaling pathways at transcriptional super-enhancers. Mol. Cell.

[9] Dawson M.A., Gudgin E.J., Horton S.J., Giotopoulos G., Meduri E., Robson S., Cannizzaro E., Osaki H., Wiese M., Putwain S. (2014). Recurrent mutations, including NPM1c, activate a BRD4-dependent core transcriptional program in acute myeloid leukemia. Leukemia.

[10] Hnisz D., Abraham B.J., Lee T.I., Lau A., Saint-Andre V., Sigova A.A., Hoke H.A., Young R.A. (2013). Super-enhancers in the control of cell identity and disease. Cell.

[11] Zhou H., Schmidt S.C., Jiang S., Willox B., Bernhardt K., Liang J., Johannsen E.C., Kharchenko P., Gewurz B.E., Kieff E. (2015). Epstein-Barr virus oncoprotein super-enhancers control B cell growth. Cell Host Microbe.

[12] Groschel S., Sanders M.A., Hoogenboezem R., de Wit E., Bouwman B.A., Erpelinck C., van der Velden V.H., Havermans M., Avellino R., van Lom K. (2014). A single oncogenic enhancer rearrangement causes concomitant *EVI1* and *GATA2* deregulation in leukemia. Cell.

[13] Northcott P.A., Lee C., Zichner T., Stutz A.M., Erkek S., Kawauchi D., Shih D.J., Hovestadt V., Zapatka M., Sturm D. (2014). Enhancer hijacking activates GFI1 family oncogenes in medulloblastoma. Nature.

[14] Chipumuro E., Marco E., Christensen C.L., Kwiatkowski N., Zhang T., Hatheway C.M., Abraham B.J., Sharma B., Yeung C., Altabef A. (2014). CDK7 inhibition suppresses super-enhancer-linked oncogenic transcription in MYCN-driven cancer. Cell.

[15] Vahedi G., Kanno Y., Furumoto Y., Jiang K., Parker S.C., Erdos M.R., Davis S.R., Roychoudhuri R., Restifo N.P., Gadina M. (2015). Super-enhancers delineate disease-associated regulatory nodes in T cells. Nature.

[16] Achour M., le Gras S., Keime C., Parmentier F., Lejeune F.X., Boutillier A.L., Neri C., Davidson I., Merienne K. (2015). Neuronal identity genes regulated by super-enhancers are preferentially down-regulated in the striatum of huntington’s disease mice. Hum. Mol. Genet..

[17] Chapuy B., McKeown M.R., Lin C.Y., Monti S., Roemer M.G., Qi J., Rahl P.B., Sun H.H., Yeda K.T., Doench J.G. (2013). Discovery and characterization of super-enhancer-associated dependencies in diffuse large B cell lymphoma. Cancer Cell.

[18] Brown J.D., Lin C.Y., Duan Q., Griffin G., Federation A.J., Paranal R.M., Bair S., Newton G., Lichtman A.H., Kung A.L. (2014). Nf-κB directs dynamic super enhancer formation in inflammation and atherogenesis. Mol. Cell.

[19] Creyghton M.P., Cheng A.W., Welstead G.G., Kooistra T., Carey B.W., Steine E.J., Hanna J., Lodato M.A., Frampton G.M., Sharp P.A. (2010). Histone H3K27ac separates active from poised enhancers and predicts developmental state. Proc. Natl. Acad. Sci. USA.

[20] Parker S.C., Stitzel M.L., Taylor D.L., Orozco J.M., Erdos M.R., Akiyama J.A., van Bueren K.L., Chines P.S., Narisu N., Black B.L. (2013). Chromatin stretch enhancer states drive cell-specific gene regulation and harbor human disease risk variants. Proc. Natl. Acad. Sci. USA.

[21] Ernst J., Kellis M. (2012). Chromhmm: Automating chromatin-state discovery and characterization. Nat. Methods.

[22] Ernst J., Kheradpour P., Mikkelsen T.S., Shoresh N., Ward L.D., Epstein C.B., Zhang X., Wang L., Issner R., Coyne M. (2011). Mapping and analysis of chromatin state dynamics in nine human cell types. Nature.

[23] Quang D.X., Erdos M.R., Parker S.C., Collins F.S. (2015). Motif signatures in stretch enhancers are enriched for disease-associated genetic variants. Epigenet. Chromatin.

[24] Dowen J.M., Fan Z.P., Hnisz D., Ren G., Abraham B.J., Zhang L.N., Weintraub A.S., Schuijers J., Lee T.I., Zhao K. (2014). Control of cell identity genes occurs in insulated neighborhoods in mammalian chromosomes. Cell.

[25] Yin J.W., Wang G. (2014). The mediator complex: A master coordinator of transcription and cell lineage development. Development.

[26] Kagey M.H., Newman J.J., Bilodeau S., Zhan Y., Orlando D.A., van Berkum N.L., Ebmeier C.C., Goossens J., Rahl P.B., Levine S.S. (2010). Mediator and cohesin connect gene expression and chromatin architecture. Nature.

[27] Herranz D., Ambesi-Impiombato A., Palomero T., Schnell S.A., Belver L., Wendorff A.A., Xu L., Castillo-Martin M., Llobet-Navas D., Cordon-Cardo C. (2014). A NOTCH1-driven MYC enhancer promotes T cell development, transformation and acute lymphoblastic leukemia. Nat. Med..

[28] Lee T.I., Young R.A. (2013). Transcriptional regulation and its misregulation in disease. Cell.

[29] Izumi K., Nakato R., Zhang Z., Edmondson A.C., Noon S., Dulik M.C., Rajagopalan R., Venditti C.P., Gripp K., Samanich J. (2015). Germline gain-of-function mutations in AFF4 cause a developmental syndrome functionally linking the super elongation complex and cohesin. Nat. Genet..

[30] Vissers L.E., van Ravenswaaij C.M., Admiraal R., Hurst J.A., de Vries B.B., Janssen I.M., van der Vliet W.A., Huys E.H., de Jong P.J., Hamel B.C. (2004). Mutations in a new member of the chromodomain gene family cause charge syndrome. Nat. Genet..

[31] Schnetz M.P., Handoko L., Akhtar-Zaidi B., Bartels C.F., Pereira C.F., Fisher A.G., Adams D.J., Flicek P., Crawford G.E., Laframboise T. (2010). CHD7 targets active gene enhancer elements to modulate ES cell-specific gene expression. PLoS Genet..

[32] Schnetz M.P., Bartels C.F., Shastri K., Balasubramanian D., Zentner G.E., Balaji R., Zhang X., Song L., Wang Z., Laframboise T. (2009). Genomic distribution of CHD7 on chromatin tracks H3K4 methylation patterns. Genome Res..

[33] Petrij F., Giles R.H., Dauwerse H.G., Saris J.J., Hennekam R.C., Masuno M., Tommerup N., van Ommen G.J., Goodman R.H., Peters D.J. (1995). Rubinstein-Taybi syndrome caused by mutations in the transcriptional co-activator CBP. Nature.

[34] Feng W., Khan M.A., Bellvis P., Zhu Z., Bernhardt O., Herold-Mende C., Liu H.K. (2013). The chromatin remodeler CHD7 regulates adult neurogenesis via activation of SoxC transcription factors. Cell Stem Cell.

[35] Lopez A.J., Wood M.A. (2015). Role of nucleosome remodeling in neurodevelopmental and intellectual disability disorders. Front. Behav. Neurosci..

[36] Delmore J.E., Issa G.C., Lemieux M.E., Rahl P.B., Shi J., Jacobs H.M., Kastritis E., Gilpatrick T., Paranal R.M., Qi J. (2011). BET bromodomain inhibition as a therapeutic strategy to target c-Myc. Cell.

[37] Affer M., Chesi M., Chen W.D., Keats J.J., Demchenko Y.N., Tamizhmani K., Garbitt V.M., Riggs D.L., Brents L.A., Roschke A.V. (2014). Promiscuous MYC locus rearrangements hijack enhancers but mostly super-enhancers to dysregulate MYC expression in multiple myeloma. Leukemia.

[38] Mansour M.R., Abraham B.J., Anders L., Berezovskaya A., Gutierrez A., Durbin A.D., Etchin J., Lawton L., Sallan S.E., Silverman L.B. (2014). Oncogene regulation. An oncogenic super-enhancer formed through somatic mutation of a noncoding intergenic element. Science.

[39] National Cancer Institute. www.cancer.gov/types/childhood-cancers.

[40] Puente X.S., Bea S., Valdes-Mas R., Villamor N., Gutierrez-Abril J., Martin-Subero J.I., Munar M., Rubio-Perez C., Jares P., Aymerich M. (2015). Non-coding recurrent mutations in chronic lymphocytic leukaemia. Nature.

[41] Garriga J., Grana X. (2004). Cellular control of gene expression by T-type cyclin/CDK9 complexes. Gene.

